# An integrated primary care workforce planning toolkit at the regional level (part 1): qualitative tools compiled for decision-makers in Toronto, Canada

**DOI:** 10.1186/s12960-021-00610-2

**Published:** 2021-07-21

**Authors:** Caroline Chamberland-Rowe, Sarah Simkin, Ivy Lynn Bourgeault

**Affiliations:** University of Ottawa and Canadian Health Workforce Network, Ottawa, Canada

**Keywords:** Integrated health workforce planning, Primary care, Population health needs, Regional planning, Multi-professional, Service-focused, Practice patterns, Population mobility

## Abstract

**Background:**

A regional health authority in Toronto, Canada, identified health workforce planning as an essential input to the implementation of their comprehensive Primary Care Strategy. The goal of this project was to develop an evidence-informed toolkit for integrated, multi-professional, needs-based primary care workforce planning for the region. This article presents the qualitative workforce planning processes included in the toolkit.

**Methods:**

To inform the workforce planning process, we undertook a targeted review of the health workforce planning literature and an assessment of existing planning models. We assessed models based on their alignment with the core needs and key challenges of the health authority: multi-professional, population needs-based, accommodating short-term planning horizons and multiple planning scales, and addressing key challenges including population mobility and changing provider practice patterns. We also assessed the strength of evidence surrounding the models’ performance and acceptability.

**Results:**

We developed a fit-for-purpose health workforce planning toolkit, integrating elements from existing models and embedding key features that address the region’s specific planning needs and objectives. The toolkit outlines qualitative workforce planning processes, including scenario generation tools that provide opportunities for patient and provider engagement. Tools include STEEPLED Analysis, SWOT Analysis, an adaptation of Porter’s Five Forces Framework, and Causal Loop Diagrams. These planning processes enable the selection of policy interventions that are robust to uncertainty and that are appropriate and acceptable at the regional level.

**Conclusions:**

The qualitative inputs that inform health workforce planning processes are often overlooked, but they represent an essential part of an evidence-informed toolkit to support integrated, multi-professional, needs-based primary care workforce planning.

## Background

Because a fit-for-purpose workforce is contextually determined [1], health workforce planning (HWP) must adapt the use of data, methodological approaches, interpretations, and recommendations to the realities and goals of the local system. Mixed methods approaches to HWP mobilize the strengths of both qualitative and quantitative approaches to provide decision-makers with practical recommendations, and to enable corresponding evidence-based action within the health system. Mixed methods approaches: (1) address data and methodological limitations associated with the independent use of either quantitative of qualitative methods; (2) account for the uncertainty that is inherent in health systems; (3) promote engagement with local stakeholders; and (4) foster a planning culture where policy levers are more readily deployed [2]. Planners can then leverage this culture to promote iterative planning with incremental refinement of estimates and course corrections rather than drastic and costly reforms to address a future that may never come to pass.

Inaccuracy and unreliability in health workforce projections often stem from planning exercises that simply project forward the status quo and fail to account for uncertainty and change in population health needs, workforce trends, or the environment within which they interact [3]. In order to account for uncertainty, planners should supplement data modelling and traditional quantitative forecasting with workforce intelligence and qualitative analyses in order to anticipate, and plan for, a system’s potential evolution over time [3, 4].

*Scenario analyses*, which contemplate a series of “what if” statements, are increasingly deployed to plan for uncertainty in complex adaptive health systems, assess policy alternatives, and test modelling assumptions. These analyses provide policy-makers with the ability to synthetically ‘shock’ the system in order to define optimal solutions in the pursuit of system objectives. Scenario analyses also provide an ideal opportunity to explore a range of possible future scenarios that are grounded not only in data but also intelligence informed by the experiences of patients, workers, and planners who are directly engaged with the system at hand, increasing the robustness of HWP exercises.

Stakeholder engagement throughout the workforce planning process can improve the acceptability of planned models of care, encourage buy-in, and facilitate resource mobilization and the implementation of plans [5]. Health systems are complex, adaptive, and human. Within these systems, workers, employers and system managers are active agents with considerable vested interest in the results of health workforce plans, which do not always align with one another. Planners can deploy qualitative methods that engage key stakeholders in the design, implementation, and interpretation of HWP models to enhance the political, social, and operational feasibility of workforce plans [6–8].

Within the Canadian context, the organization, administration, and delivery of healthcare services fall under provincial jurisdiction. In the province of Ontario, regional health authorities are responsible for coordinating, integrating, and funding health services at a local level. The Toronto Region (formerly the Toronto Central Local Health Integration Network) administers healthcare services for the 2.7 million individuals living in the City of Toronto, Canada’s largest city. The Toronto Region encompasses a highly urbanized metropolitan area that borders four other administrative Regions. Many non-residents who work in downtown Toronto or travel to access specialized services also utilize the primary care services available within the City of Toronto.

Rapid population growth, changing demographics, and disparities in access to integrated primary care between sub-regions within the City of Toronto, combined with concerns surrounding an impending wave of physician retirement, underlined the need for a more robust local-level planning process. Accordingly, the Toronto Region developed, with provider input, a comprehensive Primary Care Strategy which aimed to improve patient access to care, service integration, and system efficiency. The development of this strategy coincided with the passing of the *Patients First Act* in 2016*,* which added HWP planning to the Toronto Region’s mandate. As a result, the Toronto Region identified HWP as an essential input to the implementation of this Strategy and to improving access to primary care by adequately planning for current and future population health needs in the City of Toronto. Namely, the Toronto Region’s leadership and Health Analytics staff were interested in developing a more robust evidence base to support targeted resource deployment in areas of high workforce need. Accordingly, the Toronto Region contracted our team at the Canadian Health Workforce Network (CHWN) to develop an evidence-informed HWP toolkit in collaboration with their internal Health Analytics team, which had already established itself as a trusted source of evidence both within the Toronto Region and across local system stakeholders.

We aimed to develop a series of tools for integrated primary care workforce planning at the regional level that acknowledged and addressed key challenges in workforce planning and were tailored to local planning needs. In support of this objective, we conducted a targeted review of existing methods and models in HWP to synthesize leading practices in the development of a workforce planning toolkit. The resulting toolkit is a fit-for-purpose collection of qualitative, descriptive and quantitative processes to guide and support the Toronto Region in conducting health workforce planning activities.

This article is one of two that describe the co-development of an evidence-informed, fit-for-purpose, toolkit-based approach to primary care health workforce planning, guided by an overarching framework and set of key principles outlined in an introductory commentary by Bourgeault et al. [9]. This paper (part 1) describes our targeted review of leading practices in health workforce planning and presents the qualitative health workforce planning tools and processes included in the toolkit. The second paper (part 2) [10] describes the process we followed to identify the data necessary to facilitate quantitative health workforce planning and introduces the fit-for-purpose multi-component quantitative workforce planning model included in the toolkit to allow the Toronto Region to conduct needs-based planning.

## Methods

Our approach to toolkit development was informed by a participatory action research framework [11], fostering close and continuous collaboration with key local partners, including the Toronto Region leadership and analytics staff, primary care physician community leaders, and representatives from the City of Toronto. Regular contacts with these partners, along with extensive consultations with community stakeholders, were instrumental in shaping toolkit development and continue to inform the refinement of the toolkit and its outputs as we proceed with its operationalization. By prioritizing engagement with local partners and stakeholders, we sought not only to bolster the acceptability and validity of our outputs, but to build local capacity for HWP and to foster a common commitment to realizing the benefits of robust planning processes.

We undertook a targeted review of health workforce planning literature and an assessment of existing planning models. We assessed models based on their alignment with a list of guiding principles that reflected the Toronto Region’s organizational values and priorities, their operational and technical requirements, as well as the key challenges that define the context within which they operate.

We also assessed the strength of evidence surrounding the models’ performance and acceptability. This review was complemented by a concurrent scan of available quantitative datasets to inform the development of a quantitative model (see part 2 by Simkin et al. [10]). Integrating across these two exercises, we developed a fit-for-purpose planning toolkit, including qualitative HWP processes and a quantitative HWP model, for integrated, multi-professional, needs-based primary care workforce planning.

### Identification of models for assessment

Our targeted search strategy was designed to identify models for assessment and involved a total of 12 specific searches to allow for a comprehensive review of HWP approaches as they relate to the parameters set forth by our regional partners. We implemented all search strategies in PubMed, Web of Science, and SCOPUS. We exported the resulting citations to EndNote X8. We confined the search to articles published between 1997 and 2017, in English and French. In the event that searches rendered a high volume of citations, we reviewed the first 500 citations, filtered by relevance or “best match”.

**Fig. 1:**
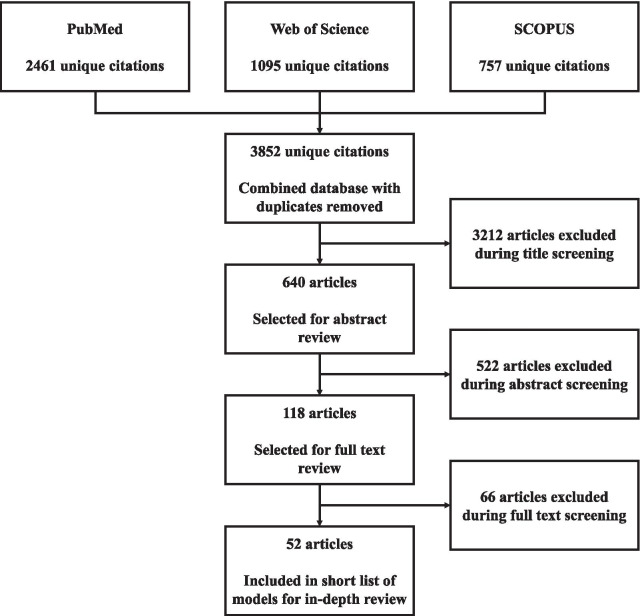
Search strategy flowchart

As depicted in Fig. 1, the 12 search strategies rendered 2461 unique citations in PubMed, 1095 unique citations in SCOPUS, and 757 unique citations in Web of Science. Following the removal of duplicates, we proceeded with a title screening of 3852 citations. Following the initial title screening, we deemed that 640 citations were eligible for abstract screening. After abstract review, 118 citations met inclusion criteria. We included articles if they presented a model for HWP that accounted for alignment between supply of and demand for health human resources, regardless of how these components were defined.

To supplement our search of academic literature, we conducted a search of grey literature on HWP. This search was particularly important given the role of public sector and multilateral organizations in HWP. We also consulted the bibliographies of existing reviews of HWP models to ensure that all relevant sources were included in our review. Our search strategy and inclusion criteria reflected an explicit focus on Canadian content, while acknowledging the opportunity to learn from leading practices in high-income and low- and middle-income countries internationally.

### Model assessment

In order to develop a ‘fit-for-purpose’ HWP toolkit for our regional partners, we used a list of guiding principles to help assess fit with the unique planning needs and objectives of the Toronto Region, based on the capacity of models to:

1. project demand as a function of population need rather than simple service utilization, to align our toolkit with the Toronto Region’s population health approach and the broader health system’s endeavor to achieve universal health coverage;
2. project alignment for individual neighbourhoods, sub-regions, and the entire City of Toronto to produce results with sufficient granularity to inform local decision-making;
3. support multi-professional or service-based, rather than uni-professional, planning, given the Toronto Region’s focus on integrated primary care;
4. provide accurate projections for short planning horizons, in light of the Toronto Region’s 1–5 year planning cycles;
5. support scenario analyses to assess the impact of changing population and provider profiles, policy interventions and modelling assumptions
6. engage primary care workers in the co-design of health workforce plans, in line with the Toronto Region’s efforts to empower and engage the primary care workforce and stakeholders in the planning process; and
7. account for key challenges in the City of Toronto, such as changing provider practice patterns and population mobility.

In their review of Health Workforce projection models deployed in OECD countries, Ono et al. [12] stated that models should be evaluated based on the process of model development, which encompasses the model’s underlying conceptual framework and variables, the performance and predictive accuracy of the model, and the acceptability and impact of the model. These criteria also informed our assessment of HWP models.

We created a literature extraction tool in Excel to systematically capture information relevant to our assessment. The tool included a row for each of the identified models, enabling the comparison of their potential contribution to HWP in the Toronto Region based on a defined list of content areas (columns). These content areas included:

- the conceptual framework employed (if any);
- the methods, variables, and data requirements for both the supply and demand components of the model;
- the model’s alignment with the key features guiding our assessment (short planning horizon, small-area planning, multi-professional planning, scenario analysis, provider engagement, practice patterns, and population mobility);
- the evidence surrounding the model’s performance and acceptability; and
- our evaluation of the strengths, weaknesses, and unique features of the model in question.

Based on the comparative analysis of the information captured in this literature extraction tool, we identified a short-list of models that were used to inform specific components of a fit-for-purpose HWP toolkit for primary care within the City of Toronto.

### Results

Tables 1, 2, and 3 present a synthesis of the models shortlisted to inform our health workforce planning process (Table 1), our service requirement and capacity projections (Table 2), and our allocation of service requirements across cadres (Table 3), respectively. These synthesis tables also describe the shortlisted models’ alignment with the needs of the Toronto Region.

**Table 1:** Synthesized assessment of shortlisted HWP models for the health workforce planning process

Models identified	Capacity for needs-based projections of service requirement	Capacity for local-level planning	Capacity to accommodate short planning horizons	Capacity for multi-professional planning	Capacity to conduct scenario analyses	Capacity to engage the workforce	Capacity to account for changing practice patterns	Capacity to account for population mobility
England’s Robust Workforce Planning Framework [13]	Uses Birch et al. [14] Needs-Based Health Human Resource Planning Framework to project service requirements	Scale defined in horizon scanning process	30-year planning horizon in 5-year increments	An additional step can be added to distribute skill hours across a chosen mix of professions using wellbeing skills cube	Uses scenarios to account for uncertainty that is inherent to health systems and uses sensitivity analysis to test impact of data variations	Elicitation of expert opinion to define sources of uncertainty, generate narrative scenarios, quantify scenario parameters, and assess the impact of policies	Includes consideration of participation rates and attrition rates for each age and gender cohort	Not addressed
New Zealand’s Workforce Intelligence and Planning Framework [15]	Integrates demographics and demand by first conducting a health needs assessment, followed by defining appropriate model of care	Can be used to inform local, regional or national-level planning	2–3 year planning horizons feed into 5–15 year plans	Amenable to multi-professional planning	Capacity for scenario analysis	Clinician and expert engagement in the environmental scanning process	Accounts for internal flows between geographic locales, institutions, sectors, and specialties	Not addressed
Australia’s Health Workforce Planning Tool [16]	Utilization-based projections	Defines a common national approach to prioritize coherence and consistency at the national level	Plans through 2025	Conducts separate exercises for doctors, nurses, and midwives using the same modelling methodology	Allows for scenario analysis to assess the impact of policy options and conduct sensitivity analysis	Consults with expert reference groups, workforce participants, clinical leads throughout the planning process	Attributes exit rates to each 5-year age and gender cohort	Not addressed

**Table 2:** Synthesized assessment of shortlisted HWP models for the service requirement and capacity projections

Models identified	Capacity for needs-based projections of service requirement	Capacity for local-level planning	Capacity to accommodate short planning horizons	Capacity for multi-professional planning	Capacity to conduct scenario analyses	Capacity to engage the workforce	Capacity to account for changing practice patterns	Capacity to account for population mobility
Canadian Institutes for Health Information Population Grouping Methodology [17]	Service requirements predicted as a function of demographic and clinical profiles of individual patients	Data outputs are at the level of the individual, and can be aggregated to a variety of planning levels/regions	Single-year projection that can be run as a time series to project further	Projects service requirements for primary care physician visits	Not addressed	Not addressed	Not addressed	Not addressed
Needs-Based Health Human Resource Planning Framework [14]	Projects need as a function of a population’s demographic and epidemiological profile, a determined level of service, and a productivity function	Has been applied at provincial and national levels, but authors claim that it can be applied to any jurisdiction	Yearly projections over a determined period	Can produce separate estimates for any provider group	Allows for scenario analysis of policy options, as well as sensitivity analysis	Not addressed	Incorporates activity and participation rates that can vary over time for each and sex cohort	Not addressed
Service and Competency-Based Health Workforce Planning [8]	Projects need as a function of a population’s demographic and epidemiological profile, a determined level of service, and a productivity function	Used at the regional level	Describes current alignment	Accounts for all professions involved in the provision of identified competencies and/or services	Uses scenarios to assess gaps based on differing rates of prevalence	Workshops to validate competency list, identify relevant scopes of practice, and determine proportion of patients requiring each competency	Incorporates activity and participation rates	Not addressed
Manitoba’s Needs-Based Planning for Generalist Physicians [18]	Compares actual utilization rates with number of visits needed, which is projected as a function of age, sex, health-related indicators, and socioeconomic characteristics	Data collected for 54 service areas and aggregated into 4 regions	Describes current alignment	Output is an aggregate of required physician visits, which encompasses general practitioners, general internist, and general paediatrician	Not addressed	Not addressed	Accounts for variation in average visit workload across regions	Produces an estimate of visit requirements generated by residents and non-residents who access care within a region while accounting for the proportion of care that each of these populations seek elsewhere

**Table 3:** Synthesized assessment of shortlisted HWP models for the allocation of service requirements across cadres

Models identified	Capacity for needs-based projections of service requirement	Capacity for local-level planning	Capacity to accommodate short planning horizons	Capacity for multi-professional planning	Capacity to conduct scenario analyses	Capacity to engage the workforce	Capacity to account for changing practice patterns	Capacity to account for population mobility
Adjusted service target-based planning [19]	Identifies the need for services based on the incidence and prevalence of health problems, demographic characteristics of the population, and service targets	Can be conducted at all levels	Can describe current alignment or use population projections to project future service requirements	Designed for multi-professional planning; projects for all professions with relevant scopes of practice that are involved in the provision of the targeted package of services	Can be run using a baseline “status quo” scenario and alternative scenarios to assess the potential impact of labour market interventions	Engagement with workers and experts to develop the planning methodology, define time allocated to each task, and to account for contextual factors in the process of allocation	Addresses overlap between scopes of practice and can account for proportion of time dedicated to non-clinical and alternative clinical activities	Not addressed
Plasticity matrices	Utilization-based	Can be conducted at multiple geographic levels (including local)	Can describe current alignment or produce prospective estimates	Designed for multi-specialty physician planning and can be applied for multi-professional planning; uses the concepts of within specialty, and between specialty plasticity	Projects under a variety of scenarios and incorporates visualization features to assess impact of policy scenarios	Clinical advisory board and technical experts provide input throughout model development	Concept of plasticity predicates that individual physicians within the same specialty may provide different scopes of service, while the scope of service of physicians in different specialties may overlap	Not addressed
Linear programming [20]	Combines oral health needs and utilization	Conducted in one regional health authority that comprises 5 subregional authorities; projections of need are produced at the level of the subregion and amalgamated to the regional level	Produces 5-year projection, but can be used descriptively	Use of linear programming to explore optimization of skill mix between dentists, dental nurses, dental therapists, and dental hygienists	Explores future scenarios for the use of skills within a dental team to inform dental therapy training	Consults an expert steering committee to define scenarios and assess the maximum proportion of care that could be undertaken by dental therapists rather than dentists	Incorporates the prevalence of part-time work in the dental therapist workforce into scenarios	Not addressed

Based on the findings of our model assessment, we developed a hybrid HWP toolkit for primary care services. Because no single model identified through our search strategy fully accommodated the Toronto Region’s needs, we integrated key features from a number of existing approaches to develop a fit-for-purpose HWP process that aligns with the specific planning needs and objectives of the Toronto Region.

The overarching HWP process that we recommended to the Toronto Region combines promising elements from three distinct HWP frameworks. England’s Robust Workforce Planning Framework [13] informed the recommended process for health workforce planning and scenario development. Australia’s Health Workforce Planning Tool [16] informed the recommended process for stakeholder and workforce engagement. Finally, our recommended workforce planning process integrates a number of environmental scanning tools presented by New Zealand’s Workforce Intelligence and Planning Framework [15].

**Fig. 2:**
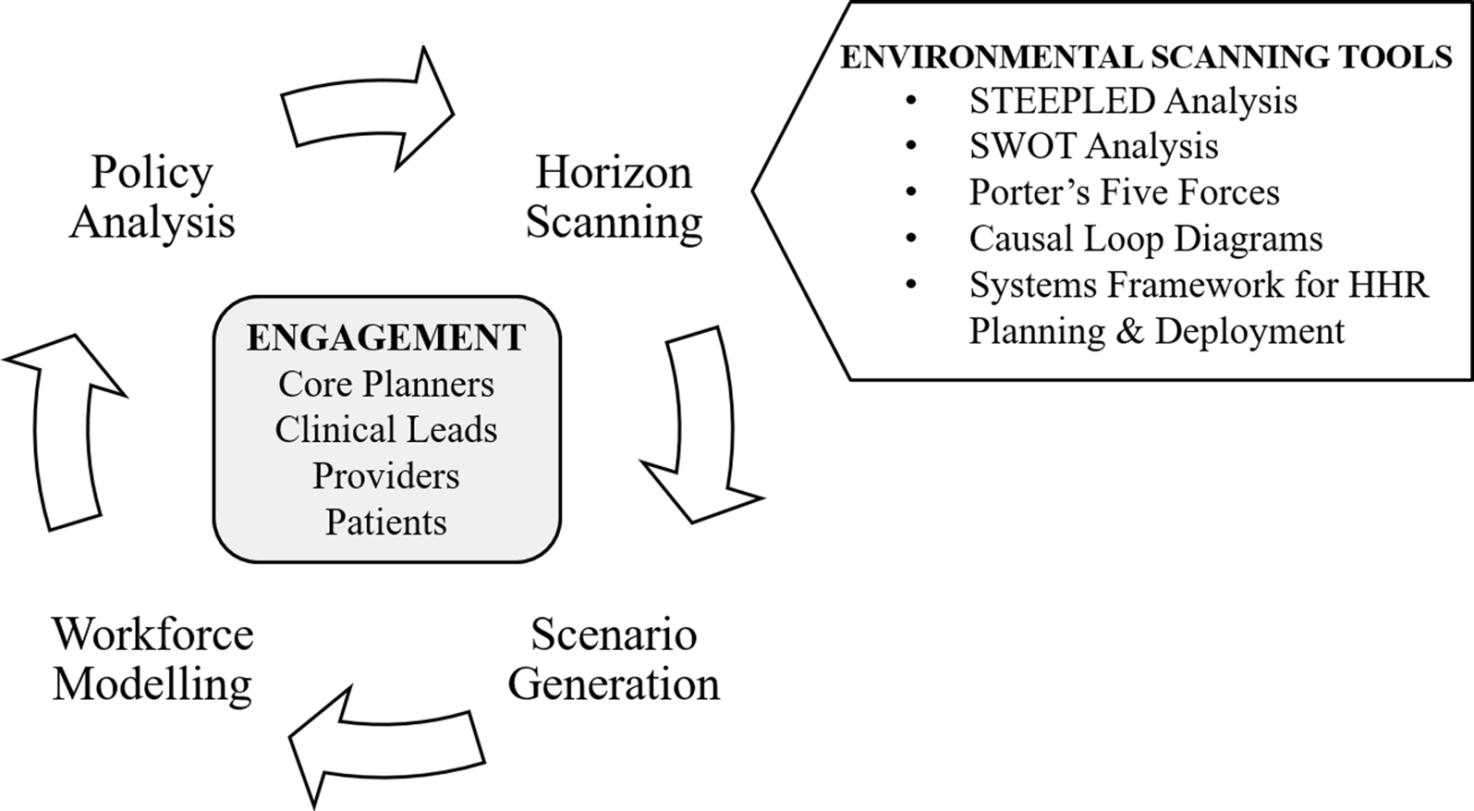
Cyclical health workforce planning process

These promising practices in HWP nest quantitative HWP models within broader health workforce and health system planning processes that are both iterative and interactive in nature. The toolkit we proposed (depicted in Fig. 2) outlines a cyclical qualitative workforce planning process that provides opportunities for primary care workforce, stakeholder, and patient engagement at all stages of planning and facilitates the evaluation and selection of policy interventions that are robust to uncertainty across a range of possible futures. While the four phases of this planning cycle—including horizon scanning, scenario generation, workforce modelling, and policy analysis—are presented in a stepwise fashion, this toolkit is explicitly iterative, encouraging planners to move back and forth between these phases in order to incrementally refine and adjust their estimates based on emerging trends, feedback from stakeholders, and ongoing assessments of the accuracy and validity of model estimates.

### Horizon scanning

The cyclical workforce planning process presented in England’s Robust Workforce Planning Framework [13] begins with a horizon scanning exercise to map the driving forces present within the system. Within the context of the Toronto Region, we have recommended that an internal planning group engage in a 1-day horizon scanning workshop using the environmental scanning tools presented by Health Workforce New Zealand [15, 21] to identify driving forces that could influence workforce and population health trends over the defined planning period.

Planners can use STEEPLED analyses^1^ (social, technological, economic, environmental, political, legal, educational, and demographic) and SWOT analyses (strengths, weaknesses, opportunities and threats) to engage in the identification of factors that can affect the ability of a system to achieve optimal or appropriate alignment between service requirements (population health needs) and service capacity (workforce supply).

First, planners can use STEEPLED analysis to identify macro-level contextual factors that merit consideration in the HWP process due to their potential impact on the health workforce or on population health and demography within a particular region. As a means of enriching discussions surrounding these eight categories of factors, we encourage planners to refer to a systems framework for HWP, and employed an example specific to the Canadian context [22]. By consulting such a framework, planners can ensure that their discussions account for the complex network of system-level inputs and policy levers that must be mobilized in order to allow for population health needs to serve as the drivers of health workforce planning and deployment.

Second, SWOT analyses allow planners to categorize external (contextual) and internal (organizational) factors as either favourable or unfavourable to the desired system outcome (e.g., a balance of population health needs and health workforce supply and capacity), and to the ability of planners to achieve this outcome through targeted planning and intervention. As an initial step for SWOT analysis, planners can categorize the contextual factors identified through the STEEPLED Analysis as either opportunities or threats. Planners can then identify internal organizational factors that should be considered in the workforce planning process and categorize them as either strengths or weaknesses.

These analytical tools allow planners to account for their sphere of influence and the policy levers at their disposal to control the factors identified. Internal factors are within the planners’ sphere of influence, and so these factors are more readily reinforced or remedied, whereas planners must develop strategies to leverage external opportunities and mitigate external threats that are beyond their sphere of influence. We have recommended that planners synthesize the outputs of this horizon scanning workshop into a brief report that can serve to frame a broader consultative process.

Planners can use environmental scanning tools in the horizon scanning phase of workforce planning to explore the breadth of factors that interact within the health region as a complex adaptive system. In subsequent stages of scenario generation and policy analysis, planners can use these same tools to delve deeper into particular issues of concern in the delivery of primary care within the region. Furthermore, all of the included environmental scanning tools can be used for both internal brainstorming and external consultation and engagement throughout the HWP process.

### Scenario generation

Scenario generation allows planners to elicit, develop and focus on HWP scenarios that are relevant to their communities. The scenario generation process is also critically important to inform the ultimate data requirements for quantitative modelling. We recommended that planners conduct scenario generation workshops at the sub-region level as well as at the city-wide level, ensuring that both local and region-wide workforce issues can be addressed. These one-day workshops are designed to bring together a broad range of stakeholders to augment the list of factors generated by the horizon scanning exercise, and develop narrative scenarios shaped by the uncertainties that may influence the future state of the system [13].

Stakeholder consultation bolsters the modelling process and reinforces the relevance of its outputs [4]. Furthermore, stakeholder engagement can foster buy-in and facilitate the acceptance of projections as a trusted evidence-base for policy action [23]. To supplement the work conducted internally by the Toronto Region and infuse the scenario generation process with local workforce intelligence, we have recommended that planners invite clinical leads from each concerned primary care cadre, patient advisors, and other relevant experts to participate in scenario generation workshops.

During these workshops, participants develop narrative scenarios that describe a reference future, which is considered to be the most probable and reasonable baseline future given current trends, as well as alternative futures that reflect the potential effects of the driving forces identified during the horizon scanning workshop. In addition to the environmental scanning tools described in the previous section, planners can use causal loop diagrams during scenario generation workshops to map the complex web of interactions between factors and system components. Once the causal loop diagram has been drawn, participants are asked to elaborate on a series of narrative scenarios that describe its interactions, and their potential impact on service requirements and capacity. Causal loop diagrams can assist workshop participants in gaining a more holistic understanding of the challenge, allow them to elaborate consistent and valid narrative scenarios, and enable them to identify the quantitative variables that require manipulation to simulate this scenario using the HWP model.

The toolkit then bridges qualitative and quantitative approaches by employing the elicitation methods described by England’s Centre for Workforce Intelligence [24]—including traditional Delphi Processes, the EFSA Delphi approach, and the Sheffield Elicitation Framework—to gain expert consensus on the estimated quantitative input parameters of narrative scenarios. These inputs reflect the potential influence of these driving forces on service requirements and capacity. We recommended that the Toronto Region host an elicitation workshop to define the parameters of the reference future using the Sheffield elicitation framework, and that the parameters for alternative scenarios be elicited remotely using the EFSA Delphi Approach. Both of these approaches allow planners to define probability distributions for each elicited parameter, including upper and lower bounds of the plausible range of values, a median value, and upper and lower quartiles.

### Workforce modelling

Embedded within the proposed HWP process is a quantitative HWP model. This model brings together modules on population health profiles, spatial patterns of utilization, unmet need, and population growth to inform service requirement projections. The model also includes a workforce profiles module which informs our service capacity projections. Planners then conduct an initial assessment of alignment between service capacity and service requirements, which is supplemented by a descriptive allocation process designed to explore workforce capacity to meet population health needs under alternative models of care. This allocation process aims to optimize the distribution of service requirements across the full spectrum of cadres contributing to integrated primary care.

Three models informed our initial assessment of alignment between service capacity and service requirements in the City of Toronto: the Canadian Institutes for Health Information Population Grouping Methodology [17], the Needs-Based Health Human Resource Planning Framework [14], and Manitoba’s Needs-Based Planning for Generalist Physicians [18]. The descriptive allocation process outlined in the toolkit is inspired by adjusted service target-based planning approaches [7, 19, 25, 26]. Simkin et al. [10] present the development of the quantitative service requirement and capacity projection tools included in this toolkit.

The quantitative scenario parameters identified through the elicitation processes can be used as inputs for the modelling stage. The HWP model should be run using the reference future scenario, as well as all scenarios defined in the previous step of the workforce planning process. Planners can introduce scenarios to assess the impact of alternative population health and workforce profiles, and of alternative allocations of services across cadres with relevant scopes of practice.

### Policy analysis

Finally, planners can hold structured workshops to explore potential policy interventions that could be conducive to remedying any misalignments highlighted by the model’s gap analysis.

We have recommended that the Toronto Region invite the expert participants who were engaged in scenario generation, and a broader range of primary care workers and patients, to participate in these discussions.

Planners can develop the narrative description and quantitative input parameters for identified policy scenarios using the tools prescribed for scenario generation. The influence of potential policy interventions can then be measured against all identified scenarios, which represent a number of potential futures. Policies are therefore considered “robust” to uncertainty if they produce favourable workforce outcomes against a high proportion of potential futures [13].

As an additional layer of robustness, Porter’s Five Forces Framework can be used to identify key forces with the potential to influence the implementation of proposed workforce policies and interventions. Planners are encouraged to assess whether the implementation of an intervention could be influenced by the bargaining power of suppliers and buyers or pose a threat to the existing workforce through the introduction of new entrants or substitutes. This framework is particularly amenable to the identification of dynamic interactions between actors and interests within health systems that could influence the implementation of proposed workforce policies and interventions. These considerations are salient given the social and political context within which HWP occurs. HWP should not only be regarded as a technical process, but also as a process that informs change to systems, organizations, and models of care that reflect embedded social and political values [19]. In developing scenarios and interpreting health workforce projections, planners must take into account the whole picture, acknowledging that political and social contexts can influence the levers at their disposal and their capacity to act upon the evidence generated by these models in order to achieve desired outcomes. By incorporating the identification of potential sources of opposition and external threats into the planning process, this toolkit enables planners to proactively address potential concerns and adapt their approach to promote the feasibility of the resulting plans.

## Discussion

### Strengths and contribution

This workforce planning toolkit pulls from extant evidence to provide planners with a fit-for-purpose approach that in this instance is tailored to the primary care planning needs of a regional health authority, but with a number of features that are transferrable to other settings. By acknowledging and leveraging the strengths of both qualitative and quantitative tools for workforce planning, this toolkit presents health workforce policy decision-makers with a comprehensive and rigorous approach to HWP. Our participatory approach to toolkit development and our explicit focus on capacity-building led to the development of a suite user-friendly HWP tools that the Toronto Region is ready and able to operationalize. The toolkit is designed to inform evidence-based decision-making, allowing policy-makers to account for uncertainty and the potential impact of interventions across a range of possible futures. Furthermore, the toolkit describes an iterative and interactive workforce planning process designed to engage key stakeholders in the elaboration and validation of scenarios, embed a planning culture into the local health system, and facilitate the mobilization of available policy levers. The toolkit’s strong emphasis on stakeholder engagement improves the social, political and operational acceptability of the resulting plans, mitigates potential sources of opposition, fosters stakeholder buy-in, and facilitates resource mobilization for the implementation of these plans.

### Limitations

HWP models, and particularly qualitative planning tools, do not produce conclusive predictions. Planners should treat workforce projections as estimates of alignment between service requirements and capacity in the event that all assumptions outlined in a given scenario are fulfilled.

Changing political landscapes can impede the operationalization of health workforce planning processes, and the scale-up of these resource-intensive innovations. In the Ontario context, since the development of the toolkit, a new provincial government has taken office and is undertaking system-wide reforms. As a result, regional health authorities’ involvement in HWP is evolving. Despite these transformations, the Toronto Region, in partnership with the City of Toronto (the municipal-level governing body), has chosen to proceed with a first cycle of HWP, which is currently underway. The Toronto Region is using this first cycle of planning to engage new entities that have emerged through these reforms in the exploration of two priority scenarios. The first relates to the service requirements associated with rapid urban development and population growth. The second explores the workforce capacity implications associated with high volumes of physician retirement.

Our team has continued to adopt a participatory approach throughout this first cycle of planning in order to build internal workforce planning capacity within the Toronto Region, and enable the progressive adaptation and refinement of the toolkit throughout implementation. As the local landscape of knowledge users continues to evolve, the impetus for workforce planning continues to grow. Our toolkit has proven to be well suited to engaging with a diverse range of stakeholders and adapting to their informational needs. In fact, the results of this first cycle of planning are in high demand from new and emerging health system organizations that intend to utilize the outputs of this planning exercise to inform the development of integrated networks of health workers and organizations that are equipped to meet the primary care needs of their target populations.

Finally, this toolkit was designed for and tailored to the needs of a metropolitan regional health authority. Therefore, adaptation would be required to allow for full transferability to other regional jurisdictions. While the principles and processes we have recommended for health workforce planning are highly relevant across jurisdictions both domestically and internationally, the technical assumptions integrated into the quantitative model are context-dependent and would require revision to reflect the unique stocks, flows, and policy levers present within different systems.

## Conclusions

By integrating a targeted review of HWP literature into the toolkit development process, we sought to highlight and address key health workforce planning challenges for a regional health authority. This toolkit presents a regional planning process that mobilizes available tools to allow for integrated, multi-professional, needs-based primary care workforce planning. Furthermore, the prescribed process enables engagement with patients, stakeholders, workers, and planners who are active within the system in the generation of locally relevant scenarios and solutions. The qualitative inputs that inform health workforce planning processes are often overlooked, but they represent an essential part of an evidence-informed toolkit to support integrated, multi-professional, needs-based primary care workforce planning.

## Data Availability

Data sharing is not applicable to this article as no datasets were generated or analysed.

## Abbreviations

CHWN: Canadian Health Workforce Network
HWP: Health Workforce Planning

## Footnotes

1 While Health Workforce New Zealand uses PESTLE analysis (Political, Economic, Sociological, Technological, Legal and Environmental), we have chosen to enhance this list of contextual factors under consideration by adopting the STEEPLED framework (Social, Technological, Economic, Environmental, Political, Legal, Educational, and Demographic) presented by Johnson, Scholes & Whittington [7].
